# Multiple Apoptotic Caspase Cascades Are Required in Nonapoptotic Roles for *Drosophila* Spermatid Individualization

**DOI:** 10.1371/journal.pbio.0020015

**Published:** 2003-12-15

**Authors:** Jun R Huh, Stephanie Y Vernooy, Hong Yu, Nieng Yan, Yigong Shi, Ming Guo, Bruce A Hay

**Affiliations:** 1Division of Biology, California Institute of TechnologyPasadena, CaliforniaUnited States of America; **2**Department of Molecular Biology, Lewis Thomas LaboratoryPrinceton University, Princeton, New JerseyUnited States of America; **3**Department of Neurology, Brain Research InstituteThe David Geffen School of Medicine at the University of California at Los Angeles, Los Angeles, CaliforniaUnited States of America

## Abstract

Spermatozoa are generated and mature within a germline syncytium. Differentiation of haploid syncytial spermatids into single motile sperm requires the encapsulation of each spermatid by an independent plasma membrane and the elimination of most sperm cytoplasm, a process known as individualization. Apoptosis is mediated by caspase family proteases. Many apoptotic cell deaths in *Drosophila* utilize the REAPER/HID/GRIM family proapoptotic proteins. These proteins promote cell death, at least in part, by disrupting interactions between the caspase inhibitor DIAP1 and the apical caspase DRONC, which is continually activated in many viable cells through interactions with ARK, the *Drosophila* homolog of the mammalian death-activating adaptor APAF-1. This leads to unrestrained activity of DRONC and other DIAP1-inhibitable caspases activated by DRONC. Here we demonstrate that ARK- and HID-dependent activation of DRONC occurs at sites of spermatid individualization and that all three proteins are required for this process. dFADD, the *Drosophila* homolog of mammalian FADD, an adaptor that mediates recruitment of apical caspases to ligand-bound death receptors, and its target caspase DREDD are also required. A third apoptotic caspase, DRICE, is activated throughout the length of individualizing spermatids in a process that requires the product of the *driceless* locus, which also participates in individualization. Our results demonstrate that multiple caspases and caspase regulators, likely acting at distinct points in time and space, are required for spermatid individualization, a nonapoptotic process.

## Abstract 2

Known as executors of programmed cell death, several caspases are here shown to be involved in *Drosophila* spermatogenesis, a process that resembles in many ways the generation of individual sperm cells in mammals, including humans

## Introduction

Most, if not all, cells have the potential to carry out the apoptotic cell death program (Jacobson et al. 1997). Key players in this process are caspase family proteases. Apical caspases are activated through interactions with adapter molecules in response to death signals arising from cellular compartments such as the mitochondria and plasma membrane death receptors. These caspases transduce death signals by cleaving and activating effector caspases. Effector caspases then cleave and alter the function of a number of cellular proteins, leading to the morphological and biochemical events associated with apoptosis (Kumar and Doumanis 2000). Proteolysis is an irreversible protein modification. Therefore, caspase activation is normally kept under tight control in viable cells. However, in *Drosophila* the apoptotic effector caspase DRICE is cleaved and activated throughout the length of elongated spermatids, and testis-specific expression of the baculovirus caspase inhibitor p35 results in male sterility, despite the fact that apoptosis is not an obligate step in spermatogenesis (Arama et al. 2003). These observations demonstrate that caspase activity is important for male fertility, but leave a number of questions unanswered: For what events in spermatid differentiation are caspases required? Which caspases mediate this requirement? How are they activated and where do they act? And how do these cells avoid apoptosis?

Spermatid development in *Drosophila* takes place within a syncytium (cyst), in which 64 haploid spermatid nuclei descended from a diploid primary spermatogonial cell are connected by abundant cytoplasmic bridges (reviewed in Lindsley and Tokuyasu 1980). In mammals, similar bridges facilitate the sharing of haploid gene products between spermatids, thereby allowing spermatid development to be directed by the products of both sets of parental chromosomes (Erickson 1973; Braun et al. 1989). It is presumed that intercellular bridges play a similar role in *Drosophila*. Ultimately, these bridges must be eliminated in a process known as individualization in order to form freely swimming sperm. At the end of male meiosis, each cyst contains 64 haploid spermatids, each approximately 2 mm long, encapsulated by two somatic cyst cells. The 64 nuclei are located at the basal end of the testis, near the seminal vesicle, and the flagellar tails extend apically, throughout the length of the testis. Individualization in *Drosophila* initiates when an actin-based structure known as an investment cone assembles around each spermatid nucleus (Tokuyasu et al. 1972). These assemble into a macroscopic structure known as the individualization complex (Fabrizio et al. 1998), which moves along the length of the cyst toward the sperm tails. The individualization complex is the site at which the cyst membrane is remodeled to enclose each sperm. Cytoplasm and organelles are extruded from between the sperm tails and pushed ahead of the individualization complex, forming a visible bulge known as the cystic bulge. When the cystic bulge reaches the sperm tails, it is detached and becomes known as the waste bag (Tokuyasu et al. 1972). A similar process, involving encapsulation of syncytial spermatids within individual plasma membranes and elimination of excess cytoplasm, also occurs during mammalian spermatogenesis (de Krester and Kerr 1994). The importance of cytoplasm elimination for human fertility is suggested by the fact that many conditions or treatments resulting in infertility disrupt this process (Russell 1991; Keating et al. 1997; Akbarsha et al. 2000). Cytoplasm elimination during spermatogenesis may also represent a strategy by which male gametes eliminate cytoplasmic parasites, thereby preventing their transmission to the zygote (Randerson and Hurst 2001).

## Results

### Caspase Activity Is Required for Spermatid Individualization

**Figure 1:**
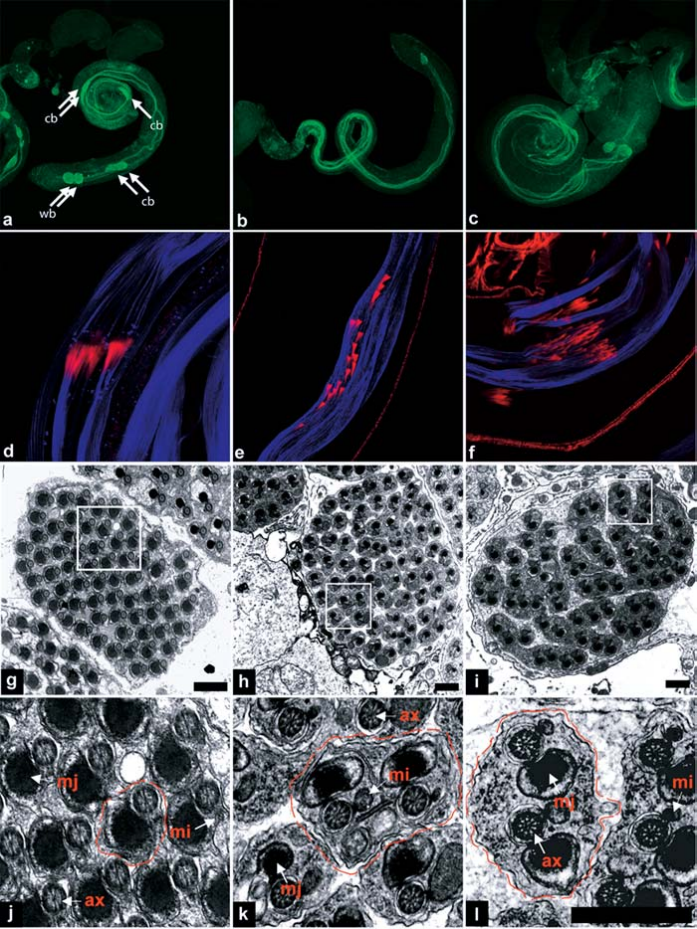
Caspase Activity Is Required for Spermatid Individualization (A–C) Testis of different genotypes were visualized with antibodies specific for activated Drice (green). (A) Wild-type testis. Active DRICE is present in multiple elongated cysts. Cystic bulges (cb) and waste bags (wb) are indicated by arrows. (B and C) Testes from β2tub-DIAP1 and β2tub-p35 males, respectively. Active DRICE is present in elongated cysts, but cystic bulges and waste bags are reduced in number and size. (D–F) Phalloidin-stained investment cones from testes of different genotypes (red). Spermatid axonemes in (D)–(F) are highlighted by the AXO49 antibody, which recognizes polyglycylated β2tub (Bressac et al. 1995) (blue). (D) In wild-type testes, investment cones move as a coordinated group. (E and F) Coordinated investment cone movement is disrupted in cysts from β2tub-DIAP1 and β2tub-p35 males, respectively. (G–L) EM sections of elongated cysts of different genotypes. (G) A cyst from a wild-type male that has undergone individualization. The boxed region is shown at higher magnification in (J), along with the locations of the major mitochondrial derivative (mj), minor mitochondrial derivative (mi), and axoneme (ax). A single spermatid unit is outlined with a dashed line. (H and I) In cysts from β2tub-DIAP1 and β2tub-p35 males, respectively, many spermatid units are present in a common cytoplasm that contains organelles, often including an enlarged minor mitochondrial derivative. Boxed regions of β2tub-DIAP1 and β2tub-p35 cysts shown in (H) and (I) are shown at higher magnification in (K) and (L), respectively. Several examples of multiple spermatids present in a common cytoplasm are outlined by the dashed line in (K) and (L). Scale bar for EM micrographs = 1 μm.

To determine whether caspase activity is required for spermatid individualization, we examined cysts from flies in which caspase activity in the male germline was inhibited. We generated flies that expressed the broad-specificity *Drosophila* caspase inhibitor DIAP1 or the baculovirus caspase inhibitor p35 under the control of the male germline-specific β_2_-tubulin promoter (β2tub-DIAP1 and β2tub-p35 flies, respectively) (Hay 2000; Santel et al. 2000). Cysts undergoing individualization contain activated versions of the effector caspase DRICE, as visualized with an anti-active DRICE-specific antibody (Arama et al. 2003). Testis from wild-type animals always contained active DRICE-positive cysts with prominent cystic bulges and waste bags (Figure 1A). In contrast, while elongated cysts from β2tub-DIAP1 and β2tub-p35 flies remained active DRICE-positive, cystic bulges and waste bags were largely absent and reduced in size when present (Figure 1B and 1C). In addition, the normally coordinated tailward movement of investment cones in active DRICE-positive wild-type cysts (Figure 1D) was dramatically disrupted in β2tub-DIAP1 males (Figure 1E). Cysts from β2tub-p35 males showed milder defects in investment cone movement (Figure 1F). These phenotypes, in conjunction with related observations by Arama et al. (2003), suggest, but do not prove, that caspase inhibition results in defects in individualization. To further test this hypothesis, we examined spermatids for individualization defects directly, using transmission electron microscopy (EM). In cysts from wild-type animals in which individualization had occurred, spermatid tails consisted largely of a flagellar axoneme and major and minor mitochondrial derivatives, all of which were tightly surrounded by a unit plasma membrane (Figure 1G and 1J). In contrast, in many cysts from β2tub-DIAP1 and β2tub-p35 flies, spermatids failed to separate from each other and contained excess cytoplasm, often including an enlarged minor mitochondrial derivative (Figure 1H and 1K and Figure 1I and 1L, respectively). Phenotypes similar to those seen in cysts from β2tub-DIAP1 and β2tub-p35 flies were also observed in cysts from flies in which levels of the caspase *Drosophila* caspase-1 (DCP-1) were decreased specifically in the male germline using RNA interference (RNAi) (β2tub-Dcp-1-RNAi flies) (Figure S1). Short prodomain caspases such as DCP-1 and DRICE are activated in response to cleavage by upstream signal-transducing caspases (Hawkins et al. 2000; Hay 2000; Meier et al. 2000; Shi 2002). Together, these observations demonstrate that caspase activity is required for individualization and suggest that DCP-1 (but perhaps not DRICE; see Discussion below) is an important downstream target caspase.

### Ark and Dronc Participate in Spermatid Individualization

**Figure 2:**
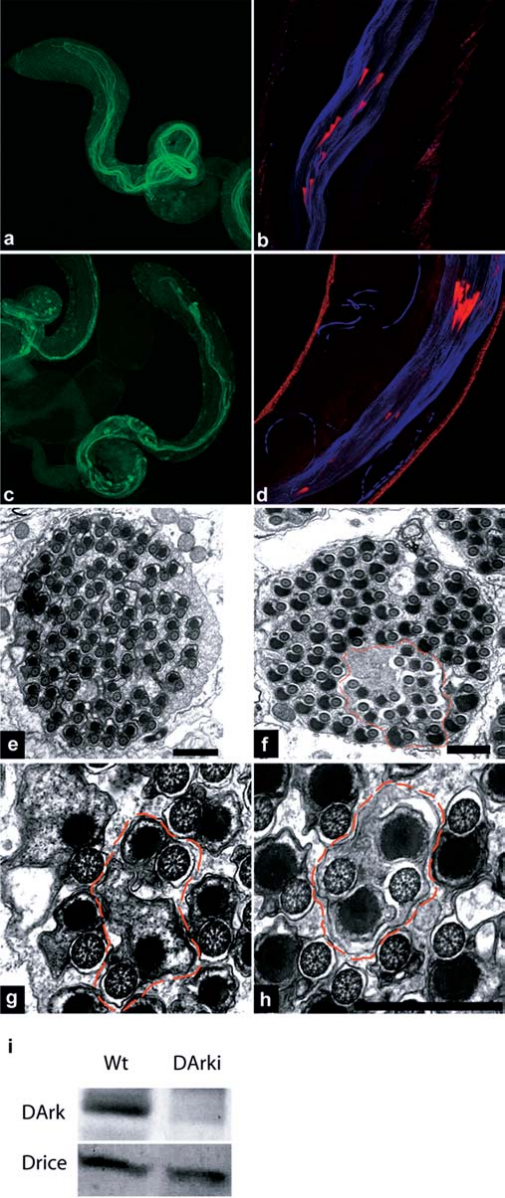
ARK and DRONC Are Required for Spermatid Individualization (A and C) Testis from β2tub-Ark-RNAi and β2tub-Dn-DRONC males, respectively. Active DRICE-positive cysts are present, but cystic bulges and waste bags are largely absent. (B and D) Investment cone movements in testis from β2tub-Ark-RNAi and β2tub-Dn-DRONC, respectively, are uncoordinated. (E, G, and H) EM images of an elongated cyst from a β2tub-Ark-RNAi male. Some individualization failures are observed (E, G, and H), two of which are highlighted by the dashed lines in (G) and (H). In addition, many spermatids that have apparently undergone individualization still contain large amounts of excess cytoplasm (E and G). (F) EM image of a cyst from a β2ub-Dn-DRONC male. A large region in which individualization did not occur is outlined. (I) Western blot from wild-type (Wt) and β2tub-Ark-RNAi (DArki) testis probed with anti-ARK and anti-DRICE antibodies. ARK, but not DRICE, levels are greatly reduced in β2tub-Ark-RNAi testis.

What are the pathways that lead to caspase activation during individualization? Cell death in many contexts in the fly requires the activity of the *Drosophila* APAF-1 homolog ARK, which promotes activation of the apical caspase DRONC (Dorstyn et al. 2002; Igaki et al. 2002; Muro et al. 2002; Zimmermann et al. 2002). DRONC, in turn, can cleave and activate the downstream caspases DCP-1 and DRICE (Hawkins et al. 2000; Meier et al. 2000; Muro et al. 2002). Genetic and biochemical evidence implicates all three of these caspases as apoptosis inducers (Kumar and Doumanis 2000). Animals homozygous for a hypomorphic *Ark* allele (*Ark^CD4^*) showed a high level of male sterility (Rodriguez et al. 1999), despite the fact that cell death is not an obligate step in spermatogenesis (Fuller 1993). This suggested to us that ARK-dependent DRONC activity might be important. To test this hypothesis, we decreased ARK levels specifically in the male germline by expressing double-stranded RNA homologous to *Ark* under the control of the β2tub promoter (β2tub-Ark-RNAi flies) (Figure 2I). To decrease levels of active DRONC, we generated flies that expressed a dominant-negative version of DRONC (Dn-DRONC) under the control of the β2tub promoter (β2tub-Dn-DRONC flies). Similar versions of DRONC are potent suppressors of DRONC-dependent cell death in other contexts (Hawkins et al. 2000; Meier et al. 2000). DRICE was still activated in elongated cysts from β2tub-Ark-RNAi and β2tub-Dn-DRONC males (Figure 2A and 2C). However, as with active DRICE-positive cysts from β2tub-DIAP1 and β2tub-p35 flies, cystic bulges and waste bags were largely absent, and coordinated investment cone movement was disrupted (Figure 2B and 2D). Examination of β2tub-ARK-RNAi and β2tub-Dn-DRONC spermatids using EM showed that inhibition of ARK (Figure 2E, 2G, and 2H) and DRONC (Figure 2F) function resulted in individualization failure in many cysts. In addition, many single spermatid units that were surrounded by a unit plasma membrane still contained large fingers of excess cytoplasm (Figure 2E and 2G). (See Discussion below.)

### ARK-Dependent Activation of DRONC Occurs at Sites of Individualization and Requires the Apoptosis Inducer HID

**Figure 3:**
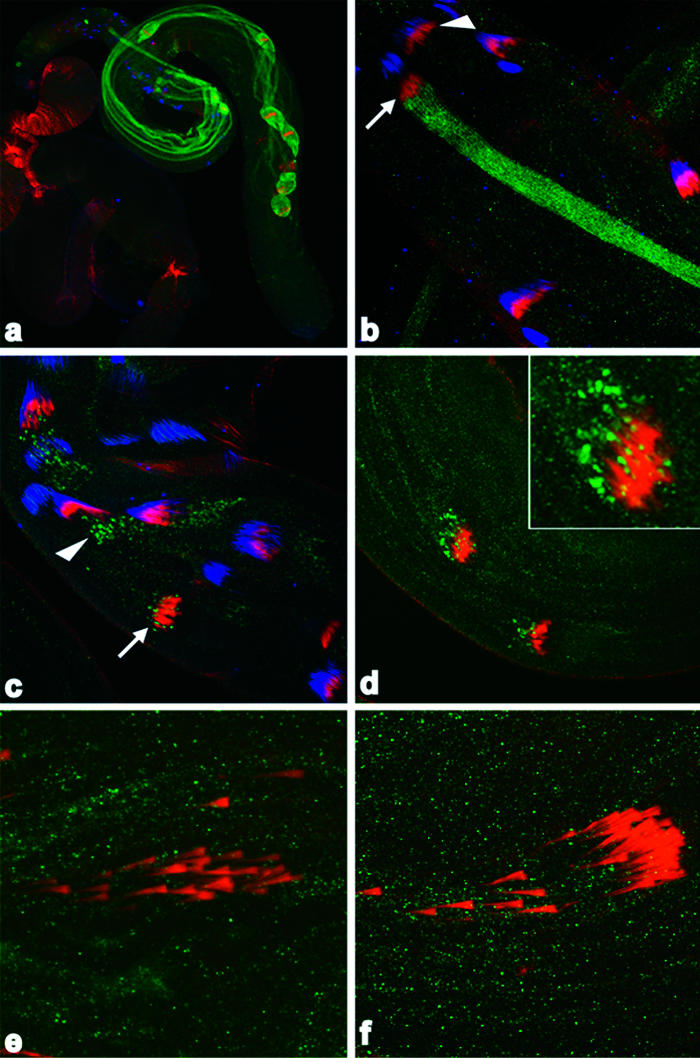
DRONC Activation Occurs in Association with Individualization Complexes and Is ARK-Dependent (A and B) Wild-type testis stained for active DRICE (green), phalloidin-stained filamentous actin (red), and TOTO-3-stained DNA (blue). (A) Active DRICE is present throughout the length of cysts undergoing individualization. (B) Higher magnification of the testis in (A). The arrowhead points to a cyst in which the individualization complex has assembled around the spermatid nuclei, but DRICE activation has not occurred. The arrow points to a neighboring cyst in which the individualization complex has just begun to move away from the spermatid nuclei. Active DRICE is now present throughout the length of this cyst, indicating that DRICE activation within a cyst occurs rapidly and globally. (C) Active DRONC (green) is initially present in a punctate pattern, apical to the individualization complex (red) at the base of the testis (arrowheads). The individualization complex then moves through the region containing active DRONC (arrow). (D) Subsequently, active DRONC is found associated with the trailing edge of the individualization complex as it moves apical within the cyst. A higher magnification view of active DRONC staining in the left-most cyst is shown in the inset. (E and F) Active DRONC is eliminated in cysts from β2tub-Ark-RNAi and β2tub-Dn-DRONC testis, respectively.

To determine where active DRONC is localized and thus where DRONC is likely to be functioning during individualization, we generated an antibody that recognized versions of DRONC that had undergone autoactivation-associated cleavage at glutamate-352 (TQT*E*) (Figure S2). In contrast to active DRICE, which appeared uniformly throughout the cyst, just as the individualization complex began its apical movement away from the spermatid nuclei (Figure 3A and 3B), active DRONC showed a dynamic pattern of localization. It was initially observed in a punctate pattern just apical to the juxtanuclear individualization complex (arrowhead in Figure 3C). The individualization complex moved through this region (arrow in Figure 3C), and active DRONC then trailed the individualization complex for the remainder of its apical movement through the cyst (Figure 3D). As expected, DRONC activation required ARK and was eliminated in testis in which ARK levels were decreased (Figure 3E) or in which access of wild-type DRONC to ARK was inhibited by expression of inactive Dn-DRONC (Figure 3F).

**Figure 4:**
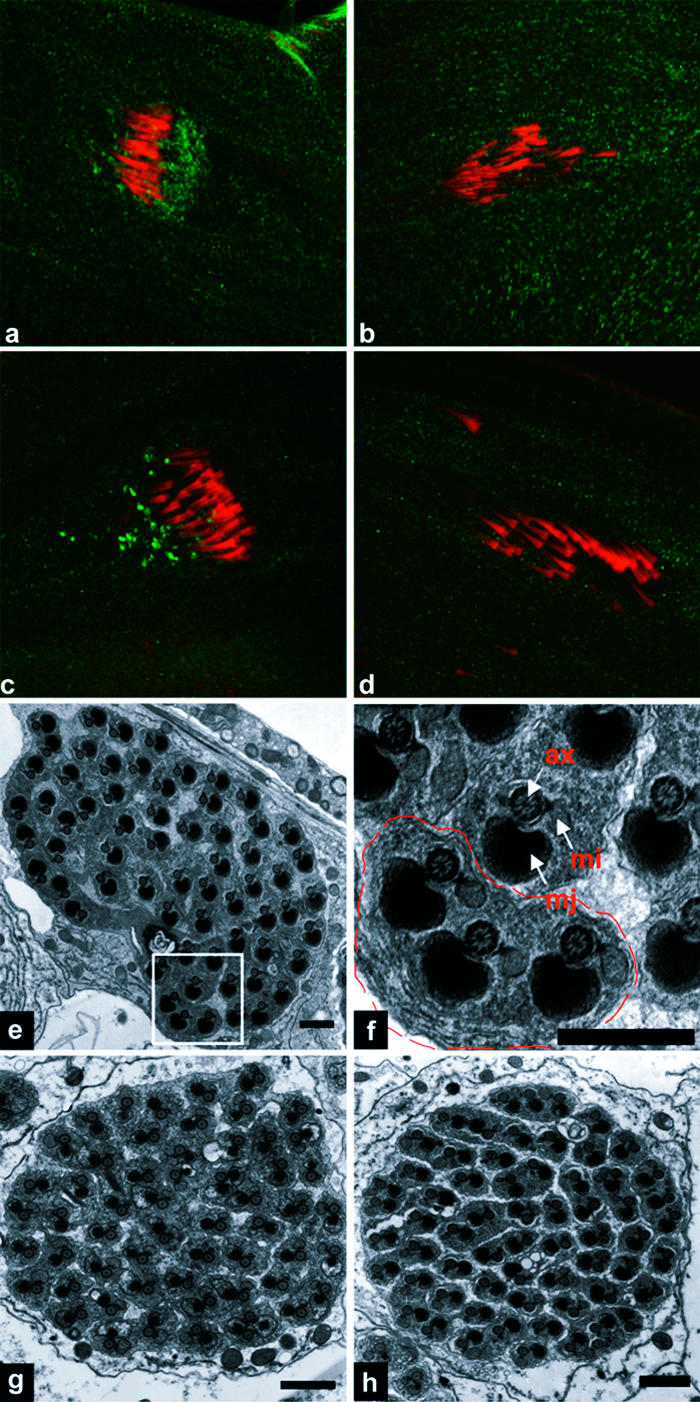
HID, dFADD, and DREDD Participate in Individualization (A) HID protein (green) is concentrated in the region of the cystic bulge, which is marked by the presence of the phalloidin-stained individualization complex (red). (B) HID immunoreactivity is absent in testis from *hid ^05014^/H99* flies. (C) Active DRONC (green) is associated with the trailing edge of the individualization complex in a wild-type cyst. (D) Active DRONC is absent from the individualization complex in cysts from *hid ^05014^/H99* males. (E) EM section from *hid ^05014^/H99* testis. Essentially all spermatids have failed to individualize. (F) Higher magnification view of boxed area in (E). Multiple spermatid units sharing a common cytoplasm are outlined by the dashed line. (G) Representative EM section of cyst from *dFadd ^f02804^*/*dFadd ^f02804^* testis. Essentially all spermatids have failed to individualize. (H) EM section of cyst from *Dredd ^B118^*/*Dredd ^B118^* testis in which individualization has failed to occur. In some other cysts from this same male, individualization proceeded apparently normally (data not shown).

How is DRONC activation during individualization regulated? DRONC undergoes continuous ARK-dependent activation in many viable cells (Dorstyn et al. 2002; Igaki et al. 2002; Muro et al. 2002; Rodriguez et al. 2002; Zimmermann et al. 2002). DIAP1 promotes the survival of these cells by ubiquitylating DRONC (Wilson et al. 2002; Chai et al. 2003) and by inhibiting the activity of caspases activated by DRONC (Hawkins et al. 1999; Wang et al. 1999). REAPER/HID/GRIM family proteins promote DRONC activity and apoptosis by disrupting DIAP1–caspase interactions, thereby preventing DIAP1-dependent ubiquitylation of DRONC and inhibition of caspases activated by DRONC (Wang et al. 1999; Goyal et al. 2000; Lisi et al. 2000; Wilson et al. 2002; Chai et al. 2003). To determine whether the REAPER/HID/GRIM family proteins played a similar role during individualization, we examined available mutants for these genes. Cysts from flies lacking *reaper* (*XR38*/*H99*) (Peterson et al. 2002) showed normal investment cone movement. In contrast, the coordinated movement of investment cones was disrupted in *hid^ 05014^*/*H99* cysts (Figure 4D, compared with Figure 4C), indicating a requirement for HID in spermatogenesis. In addition, HID protein was enriched in the cystic bulge region of wild-type cysts (Figure 4A), but not those from animals that lacked HID (*hid^ 05014^*/*H99*) (Figure 4B). These observations suggested that HID participates in DRONC activation, stabilization, or both and thereby in spermatid individualization.

Several observations support this hypothesis. First, cysts from two different *hid* allelic combinations, *hid^ A329^*/*hid^ A329^* (data not shown) and *hid^ 05014^*/*H99*, showed defects in individualization similar to those observed in β2tub-DIAP1 or β2tub-p35 males, demonstrating a requirement for HID in this process (Figure 4E and 4F). Second, localized active DRONC was eliminated in *hid^ 05014^*/*H99* (and *hid^ A329^*/*hid^ A329^*; data not shown) flies, consistent with the idea that HID promotes individualization, at least in part, by promoting DRONC activity (Figure 4D, compared with Figure 4C). HID, by virtue of its ability to disrupt IAP (inhibitor of apoptosis)–caspase interactions, may also regulate the activation of other caspase cascades during spermatid individualization (see below).

### Spermatid Individualization Utilizes Multiple Pathways of Caspase Activation

Together the above observations demonstrate that components of a canonical apoptosis-inducing pathway involving ARK, DRONC, and HID are required for spermatid individualization. However, it is important to note that the individualization defects observed in testis from β2tub-Ark-RNAi and β2tub-Dn-DRONC males (see Figure 2) were less severe then those seen in β2tub-DIAP1, β2tub-p35, or *hid^ 05014^*/*H99* males (see Figures 1 and 4). These differences may reflect incomplete inactivation of ARK and DRONC. Alternatively, they may reflect roles for ARK- and DRONC-independent caspase activities. DREDD is an interesting candidate to mediate such an activity since it is an apical caspase that can promote cell death in some contexts (Chen et al. 1998; Hu and Yang 2000). Its activation is stimulated through interactions with dFADD, the *Drosophila* homolog of mammalian FADD, an adaptor that mediates recruitment of apical caspases to ligand-bound death receptors, thereby promoting caspase activation (Hu and Yang 2000). Elongated cysts from *dFadd^ f02804^*/*dFadd^ f02804^* and *Dredd^ B118^*/*Dredd^ B118^* males (both are genetic null mutations) contained active DRICE, but often showed uncoordinated investment cone movement (data not shown). At the EM level, elongated cysts from testis of single *dFadd^ f02804^*/*dFadd ^f02804^* and *Dredd^ B118^*/*Dredd^ B118^* males showed a range of phenotypes. About 50% of cysts from *Dredd^ B118^*/*Dredd^ B118^* males and almost all cysts from *dFadd^ f02804^*/*dFadd^ f02804^* males (greater than 90%) displayed defects in individualization similar to those of β2tub-DIAP1 and β2tub-p35 flies (Figure 4G and Figure 4H, respectively). In other cysts, individualization occurred apparently normally (data not shown). Together these observations argue that dFADD and DREDD participate in individualization. The fact that loss of *dFadd* resulted in phenotypes more severe than those due to loss of *Dredd* suggests that dFADD has functions in individualization independent of promoting DREDD activation.

**Figure 5:**
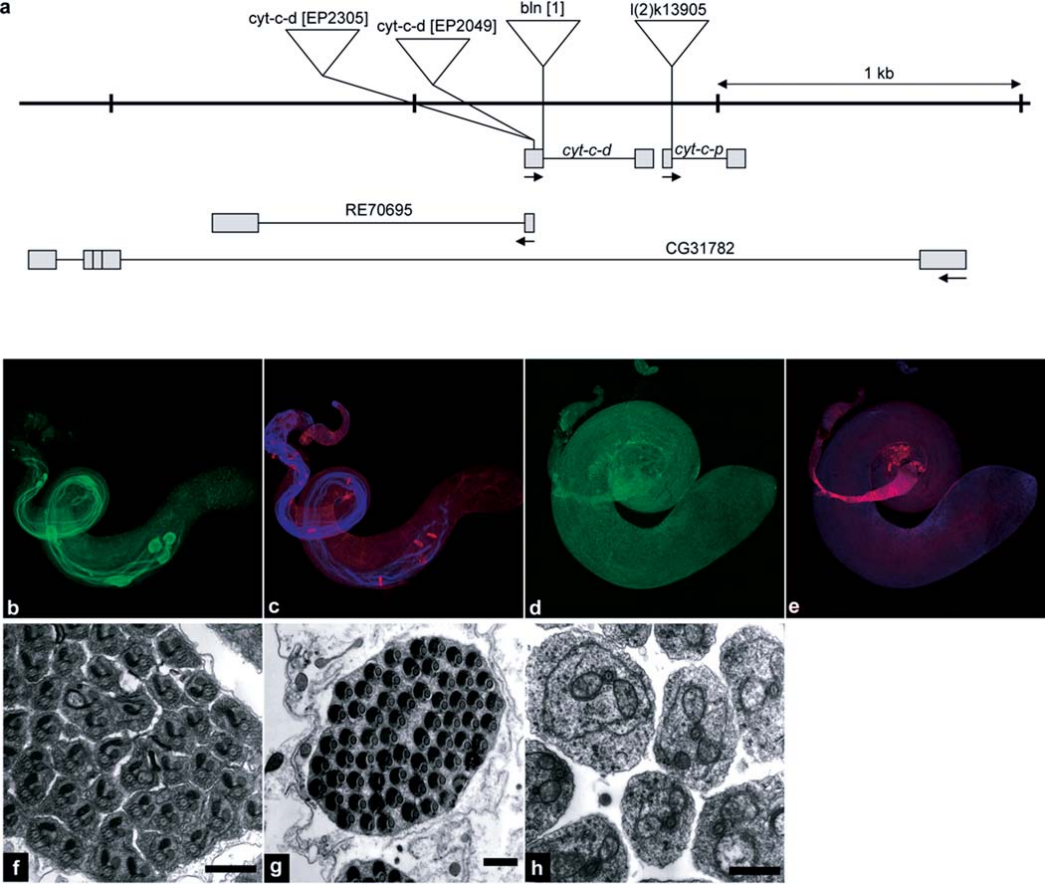
The *bln^1^* P-Element Insertion, Which Inhibits Cyt-c-d Expression, Results in Pleiotropic Defects in Spermatogenesis (A) Genomic organization of the cyt-c-d region. Upper half of the panel illustrates the structure of the region, as described by Arama et al. (2003). The lower half of the panel indicates the relative locations of several other genes in the region, as annotated by the Berkeley Drosophila Genome Project (http://flybase.bio.indiana.edu/search/) as of August 2002. The *bln^1^* P element is inserted within the cyt-c-d transcription unit. This P element is also inserted within the transcription unit of a second gene, *CR31808-RA* (*RE70695*). Both of these genes and the *bln^1^* P element reside within the intron of a third gene, *CG31782*. (B and D) Wild-type and *bln^1^* testis, respectively, stained with anti-active DRICE antibodies. Active DRICE immunoreactivity is eliminated in *bln^1^* testis, as described in Arama et al. (2003). (C and E) Wild-type and *bln^1^* testis, respectively, stained with AXO49 antibodies (blue), which recognize polyglycylated β2tub present in axonemal microtubules, and phalloidin (red). Polyglycylation occurs prior to individualization (Bressac et al. 1995). Axonemes of elongated cysts from wild-type flies stain with AXO49 (C), while those from *bln^1^* males do not (E). (F–I) EMs of cysts of different developmental stages from wild-type (F and G) and *bln^1^* (H) testis. (F) Wild-type cyst prior to individualization. Note the structures of the major and minor mitochondrial derivatives, in particular the fact that the major mitochondrial derivative is increased in size and is electron dense. (G) Wild-type cyst following individualization. (H) Representative example of the most mature cysts found in *bln^1^* testis. Note the dramatically increased cell size and the lack of differentiation of the major and mitochondrial derivatives, as compared to wild-type.

**Figure 6:**
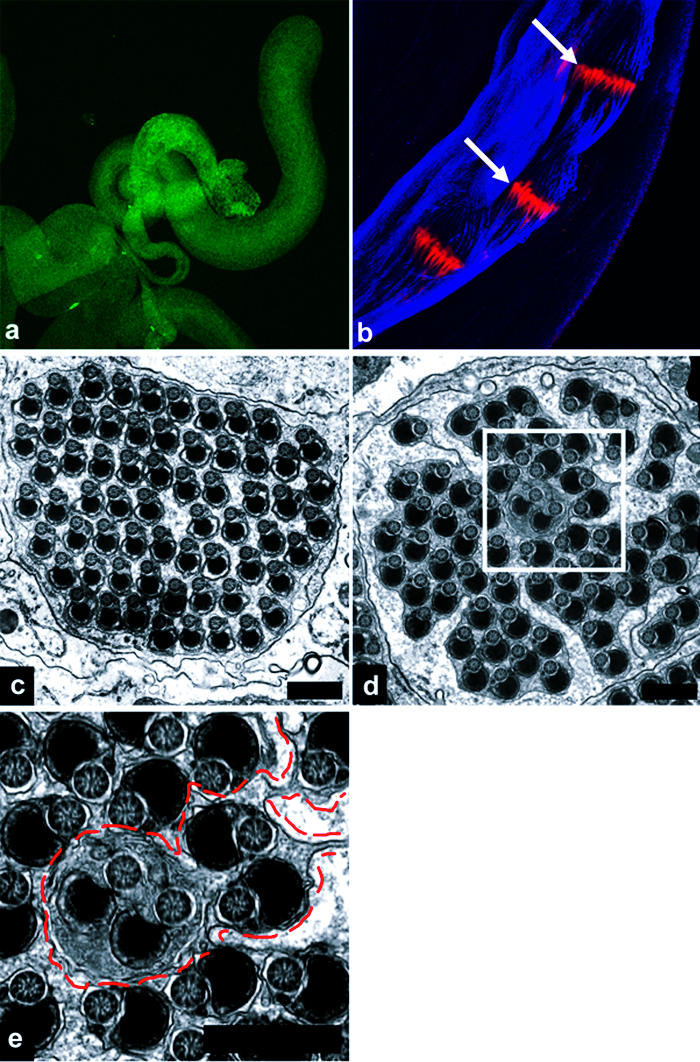
*driceless* Males Lack Active Drice Staining and Show Defects in Individualization (A) Testis from *driceless* male stained with active DRICE. Active DRICE staining is eliminated. (B) Elongated cysts from *driceless* male. AXO49 staining (blue) outlines the location of three cystic bulges. Individualization complexes (arrows) are marked with phalloidin (red). (C) Example of a cyst from a *driceless* male in which individualization has proceeded normally. (D) Example of a cyst from a *driceless* male in which individualization has failed to occur. (E) Boxed area in (D) shown at higher magnification. A region in which individualization has failed is outlined with a dashed line.

Finally, we noted that DRICE activation was insensitive to inhibition (but perhaps not to complete elimination) of ARK and DRONC; to complete loss of HID, DREDD, or FADD; and to expression of the potent general caspase inhibitors DIAP1, p35, or p49 (see Figures 1–4; data not shown). This, together with the observation that DRONC and DRICE were activated in distinct spatial and temporal patterns (see Figure 3A–3D), suggests that DRICE activation occurs through an unknown HID-, ARK-, DRONC-, dFADD-, and DREDD-independent mechanism. It has been proposed that DRICE activation in spermatids is essential for fertility and that DRICE activation is mediated by an isoform of cytochrome c, cytochrome c-d (cyt-c-d), based on the observation that males homozygous for a P-element insertion (*bln^1^*) in the *cyt-c-d* gene were sterile and lacked active DRICE staining in testis (Arama et al. 2003). However, as illustrated in Figure 5, the region surrounding the *bln^1^* insertion contains multiple transcription units. In addition, cysts from *bln^1^* males showed multiple defects in spermatogenesis prior to individualization, including failure to carry out polyglycylation of axonemal microtubules (Figure 5C and 5E), and aberrant development of the major and mitochondrial derivatives (Figure 5F–5H). These observations leave it unclear whether cyt-c-d is in any direct sense required for DRICE activation or whether DRICE is required for fertility. We serendipitously identified a line of flies carrying an X chromosome mutation (*driceless*) in which DRICE activation during spermatid individualization was completely eliminated (Figure 6A) (see Materials and Methods for details). Testis from these flies contained large cystic bulges in which individualization complexes were present as a coordinated front, as in wild-type (Figure 6B). In contrast to *bln^1^* males, *driceless* males were fertile and investment cones moved apically. As expected from this phenotype, some cysts from *driceless* males underwent individualization normally (approximately 50%) (Figure 6C). However, in others, individualization failed completely (Figure 6D and 6E).

The above observations indicate that DRICELESS promotes individualization, but leave the role of DRICE (which we have thus far been unable to effectively inactivate with RNAi) unclear. Interestingly, cysts from *driceless* males also showed reduced levels of localized active DRONC staining (data not shown), raising the possibility that DRICELESS has at least some of its effects on individualization through regulation of DRONC activity. We do not favor a simple linear model in which DRICELESS mediates its effects on individualization only by promoting DRONC-dependent activation of DRICE. This is because removal of HID or inhibition of ARK or DRONC, each of which inhibited individualization, had no significant effect on DRICE activation. An attractive alternative is that DRICELESS-dependent activation of DRICE promotes individualization, at least in part, by indirectly facilitating local activation of DRONC and perhaps other caspases, such as DREDD (see Discussion below), that are themselves activated through distinct pathways. Positive feedback pathways that perform a similar caspase-activating function have been described in a number of apoptotic contexts (Adams 2003). DRICE can cleave DIAP1 near its N-terminus. This promotes DIAP1 degradation through the N-end rule ubiquitylation pathway (Ditzel et al. 2003), providing one possible mechanism by which active DRICE could facilitate the activation of other caspases. Characterization of *driceless* should provide insight into the functional relationships between these caspases in spermatogenesis.

## Discussion

All together, our observations demonstrate that multiple caspases and caspase regulators, acting at distinct points in space and time, are utilized to promote spermatid individualization. In one pathway, whose mechanism of activation is unknown, active DRICE appears throughout elongated spermatids just as individualization begins. DRICELESS, which promotes individualization, is required for DRICE activation. But whether active DRICE mediates the requirement for DRICELESS is unknown. In a second pathway, HID, concentrated through unknown mechanisms in the cystic bulge, promotes the local ARK-dependent activation of the apical caspase DRONC, presumably at least in part through disruption of complexes between DRONC and DIAP1. As discussed above, active DRICE may facilitate this activation. Components of a second pathway for apical caspase activation, dFADD and DREDD, are also important for individualization. These proteins bind each other (Hu and Yang 2000; Horng and Medzhitov 2001), and dFADD expression promotes DREDD activation (Hu and Yang 2000). Adaptors such as mammalian FADD mediate recruitment of apical caspases to ligand-bound death receptors, thereby promoting caspase activation. Interestingly, dFADD and DREDD are absolutely required for the innate immune response to gram-negative bacterial infection (Hultmark 2003). In this pathway, dFADD-dependent activation of DREDD promotes cleavage and activation of the transcription factor RELISH. DREDD activation is mediated by homophilic death domain interactions between dFADD and IMD (an immune deficiency gene) that occur downstream of the peptidoglyclan recognition protein PGRP-LC receptor binding to bacterial cell wall components (Hultmark 2003). Homophilic death domain interactions also mediate binding of dFADD to the adaptor dMyD88, a component of the Toll receptor-dependent immune response to fungal infection (Horng and Medzhitov 2001). It will be interesting to determine whether these or other receptor pathways mediate the requirements for dFADD and DREDD during spermatid individualization.

How do caspases contribute to spermatid individualization? Testis from flies mutant for any one of the above pathways (*Ark*, *Dronc*, and *Hid*; *dFadd* and *Dredd*; and *Driceless*) contained cysts in which individualization failed to occur. Interestingly, however, other cysts in the same testis, or from testis of sibling males, carried out individualization apparently normally. Thus, these flies were fertile, though in some cases at a reduced frequency (β2tub-Ark-RNAi and *dFadd^ f02804^*/*dFadd^ f02804^*; *hid* mutants have defects in external genitalia that prevent mating). These observations suggest that no one of these caspase pathways is absolutely required for individualization. The stochastic nature of the defects observed complete failure of individualization in some mutant cysts and apparently normal individualization in others may reflect a requirement for a threshold level of caspase activity, which can be achieved through multiple pathways, or as a result of positive feedback between pathways, in order for a cyst to initiate individualization. Consistent with these possibilities, double mutants between components of the *Ark* and *Dronc* caspase cascade and mutants in the *dFadd* and *Dredd* cascade were almost completely sterile (*Dredd^ B118^*/*Dredd ^B118^*; *Ark^CD4^*/*Ark^CD4^*, 8% fertile, *n* = 12) or completely sterile (*Ark^CD4^*/*Ark^CD4^*; *dFadd ^f02804^*/*dFadd^ f02804^*, *n* = 12), while single mutants for any of these components showed significant fertility (*Dredd^ B118^*/*Dredd^ B118^*, 79% fertile, *n* = 24; *Ark^CD4^*/*Ark^CD4^*, 70% fertile, *n* = 20; *dFadd^ f02804^*/*dFadd^ f02804^*, 71% fertile, *n* = 14).

**Figure 7:**
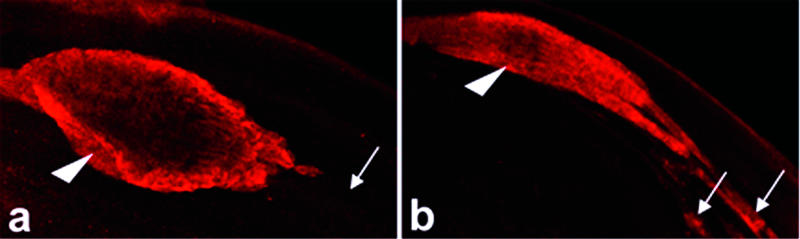
Active DRICE Is Eliminated from the Cytoplasm of Wild-Type Spermatids Following Passage of the Individualization Complex, but Not from Spermatids in Which Caspase Activity Has Been Inhibited (A) Cystic bulge from a wild-type cyst stained with active DRICE (red). The cystic bulge (arrowhead) is moving to the left. Active DRICE staining is absent in areas of the spermatid bundle that the individualization complex has passed through and in which excess cytoplasm has been eliminated (arrow). (B) Cystic bulge from a β2tub-p35 cyst. The cystic bulge (arrowhead) is decreased in size, and active DRICE is present in areas of the spermatid bundle through which the individualization complex has moved (arrows). These observations suggest caspase inhibition results in at least a partial failure to eliminate excess cytoplasm, but that this is not necessarily associated with lack of movement of the individualization complex.

Caspase activity may also participate more directly in processes that mediate encapsulation or cytoplasm elimination. Several observations suggest a role for caspases in at least the latter process. First, in contrast to the situation in wild-type cysts, active DRICE was not effectively swept up into the stunted cystic bulges formed in the presence of caspase inhibitors such as p35 (Figure 7) or in other contexts in which caspase activity was inhibited (β2tub-DIAP1, β2tub-Dcp-1-RNAi, β2tub-Ark-RNAi, β2tub-Dn-DRONC, *hid^ 05014^*/*H99*, *dFadd^ f02804^*/*dFadd^ f02804^*; data not shown). Second, spermatids in cysts with decreased levels of ARK often contained large fingers of excess cytoplasm despite the fact that in some cysts membrane encapsulation occurred apparently normally (see Figure 2). Together these observations are interesting because they also suggest that the processes of investment cone movement and spermatid encapsulation can be separated from that of cytoplasm elimination. Investment cones carry out a daunting task. They move apically within a cyst for more than 2 mm, sieving and sweeping an ever-increasing body of cytoplasmic organelles, components of the nuclear membrane, nucleoplasm, and bulk cytoplasm in front of them. Little is known about how investment cones function other than that movement is actin-based and that a number of actin-binding proteins are located in or around these structures (Hicks et al. 1999; Noguchi and Miller 2003). It is tempting to speculate that spermatid caspase activity functions, at least in part, to free organelles from preexisting attachments, thus facilitating their apical transport. In this way, caspase activity would provide a permissive environment for investment cone movement and cytoplasm removal. More active roles in promoting membrane remodeling or investment cone-dependent force generation or movement, based on spatially restricted cleavage of cytoskeletal components or other proteins, can also be imagined. The identification of caspase substrates will be important in understanding how caspases regulate this process.

What is the relationship of our observations in *Drosophila* to spermatid differentiation in mammals? During step 18 of murine spermatid differentiation, a lobe of cytoplasm accumulates around the spermatid head. It then separates from the spermatid body and is ultimately phagocytosed by the associated Sertoli cell (de Krester and Kerr 1994). Separation of this mass, known as the residual body, removes a large volume of spermatid cytoplasm. It also brings about the encapsulation of each spermatid within a single plasma membrane, since the cytoplasmic bridges linking spermatids are between the membrane compartments defined by the residual bodies. Finally, it severs the connection between the spermatid and the Sertoli cell that supported and anchored it, thereby freeing the now-individualized spermatozoa to enter the seminiferous tubule. Residual bodies show several features commonly associated with apoptosis: their plasma membrane binds Annexin V, and they are phagocytosed by Sertoli cells (Blanco-Rodriguez and Martinez-Garcia 1999), which also phagocytose apoptotic germ cells (compare Shiratsuchi et al. 1997 and references therein). In addition, residual body cytoplasm is condensed and contains elevated levels of CASPASE-1 (Blanco-Rodriguez and Martinez-Garcia 1999) and the proapoptotic BCL-2 family member BAK (Krajewski et al. 1996). These observations suggest that, as in *Drosophila*, local activation of apoptotic caspase cascades within late-stage spermatids promotes their individualization and elimination of excess cytoplasm. Mice lacking the proapoptotic proteins APAF-1 or the BLC-2 family member BAX are infertile and have dramatic defects in spermatogenesis (Knudson et al. 1995; Honarpour et al. 2000; Russell et al. 2002). However, these phenotypes are thought to be an indirect consequence of a failure in an earlier, normally occurring postnatal spermatogonial cell death. A test of the importance of caspase activity in mammalian spermatid differentiation will be most directly achieved by determining the consequences of caspase inhibition specifically in these cells.

Finally, how is it that elongated spermatids avoid apoptosis in the presence of activated apoptotic caspases for prolonged periods of time? Perhaps the caspase substrates are different from those targeted during apoptosis. But, if so, then what is the basis for the selective targeting? If the targets are the same as those activated during apoptosis, then how is the caspase cascade kept from promoting an apoptotic cell fate? Tight control over the subcellular site of caspase activation (or stabilization of the active caspase), such as we observed with DRONC, provides one possible solution. Others may also exist. In particular, it is important to recognize that while active caspase-specific antibodies recognize caspases that are in the cleaved and therefore activated conformation, these caspases may be kept inactive through interactions with other proteins or as a result of posttranslational modification. *Drosophila* is a powerful system in which to isolate male-sterile mutants (compare Castrillon et al. 1993; Fuller 1993; Fabrizio et al. 1998). It is likely that an exploration of the relationship between the genes identified by these mutations and the apoptotic regulators described here will provide insight into these questions.

## Materials and Methods

### 

#### Fly strains and constructs

All crosses and stocks were maintained at 25°C. The following fly stocks were used: *w1118, Ark^CD4^/Cyo* (Rodriguez et al. 1999), *H99/TM3* (White et al. 1994), *hid^05014^/TM3* (Grether et al. 1995), *dFadd ^f02804^/TM6B* (Naitza et al. 2002), *Dredd ^B118^/FM7* (Leulier et al. 2000), *GMR-Dronc^ F118E^* (Chai et al. 2003), and *bln^1^/Cyo* (Castrillon et al. 1993). *Dronc^ F118E^* contains a mutation that prevents interaction between DRONC and DIAP1. Thus, *Dronc ^F118E^* has enhanced activity in vivo (Chai et al. 2003). The P-element vector pβ2Tub contains sequences from the β2tub locus (85D) sufficient to direct testis germline-specific expression. It was generated by removing an XhoI–EcoRI promoter fragment from pGMR (Hay et al. 1994) and introducing in its place a 340-bp fragment from the β2tub locus (Santel et al. 2000), amplified by PCR with the primers 5′-gcg ctc gag atc ctc tat tgc ttc caa ggc acc and 5′-gcg gaa ttc agt tag ggc ccc ttt ttc aca ccg. Coding region fragments for Dn-DRONC (Hawkins et al. 2000) and DIAP1C422Y (which results in stabilization of DIAP1 by blocking its ability to autoubiquitinate [Yoo et al. 2002]) were introduced into pβ2Tub to produce pTub-Dn-DRONC and pTub-DIAP1, respectively. A vector to express double-stranded RNA for ARK was generated as follows. A 900-bp fragment of *Ark* genomic DNA corresponding to the first exon and intron was amplified using primers 5′-gcg gaa ttc ccg aag agg cat cgc gag cat ata cg and 5′-cgc aga tct ata agg ggt gag tgc tcc cag cgg ctc. This was introduced into pβ2Tub using EcoRI and BglII. A second fragment corresponding to the first exon, but in reverse orientation, was amplified using primers 5′-gcg gcg gcc gc gct aac gca ggg tcc ttc gga ggc and 5′-cgc agg cct aag agg cat cgc gag cat ata cgc. This was introduced into the intermediate described above using NotI and StuI, generating pTub-Ark-RNAi. A similar strategy was used to generate pTub-Dcp-1-RNAi. A 540-bp fragment of *Dcp-1* genomic DNA corresponding to the first exon and intron was amplified using primers 5′-ctg ccg gaa ttc ttc gac ata ccc tcg ctg and 5′-cgc gga aga tct gtt gcg cca gga gaa gta g. A second fragment corresponding to the first exon, but in reverse orientation, was amplified using primers 5′-aag gaa aaa a gcg gcc gc cgg aat ggt cga gta gga gaa g and 5′-cgc gga agg cct ttg aaa acc tgg gat tc. Germline transformants of pTub-Dn-DRONC, pTub-DIAP1, and pTub-Ark-RNAi were created using standard procedures. Testis characterized in this paper carried multiple copies of the relevant β2tub expression transgene. These were β2tub-DIAP1, β2tub-p35, and β2tub-Dn-DRONC (four copies); β2tub-Ark-RNAi (three copies); β2tub-Dcp-1-RNAi (six copies).

#### Isolation of the *driceless* mutant

We stained testis from *puc^ E69^/TM6B* males (Martin-Blanco et al. 1998) with active DRICE antibodies. These males lacked active DRICE staining, but fully elongated axonemes were present, as visualized by staining with AXO49 antibody. The mutation was mapped to the X chromosome using standard procedures.

#### Immunocytochemistry

Conditions for immunocytochemistry and confocal microscopy were as described in Yoo et al. (2002). Palloidin-Alexafluor488 (Molecular Probes Inc., Eugene, Oregon, United States) was used at 1:40 concentration to label filamentous actin; TOTO-3 was used for DNA labeling at 1:10,000 (Molecular Probes Inc.). Antibodies were used at the following concentrations: purified rabbit anti-active DRICE (1:50) (Yoo et al. 2002); purified rabbit anti-DRONC (1:100) (this paper); mouse anti-DIAP1 (1:400) (Yoo et al. 2002); mouse anti-AXO49 (1:5,000) (Bressac et al. 1995), rabbit anti-HID (1:1,000) (Yoo et al. 2002), and purified rabbit anti-active DRONC peptide (1:50) and anti-DCP-1 (1:100) (this paper). Anti-DCP-1 antibodies were produced in rabbits and purified using a C-terminal 6× His-tagged version of the DCP-1 p20 subunit as the immunogen. Anti-DRONC antibodies were raised against the C-terminal fragment of the DRONC large subunit (amino acid residues 336–352; EPVYTAQEEKWPDTQTE), and anti-active DRONC-specific antibodies were raised in rabbits using a synthetic nonapeptide corresponding to residues just N-terminal to the DRONC autoactivation cleavage site E352 (EKWPD*TQTE*), both of which were conjugated with keyhole limpet hemocyanin as the immunogen (Covance Research Products Inc., Richmond, California, United States). Active DRONC-specific antibodies were purified by sequential protein affinity purification. Antisera were first applied to a column bound with full-length inactive DRONC (*Dronc^C318A^*) to eliminate antibodies reactive with uncleaved DRONC. The flowthrough was applied to a DRONC large subunit (residues 1–352) affinity column. Bound proteins were eluted using 100 mM glycine (pH 2.5). These antibodies detect the large fragment of active DRONC (cleaved after E352), but do not recognize full-length DRONC (see Figure S2). Anti-DRONC antibodies were purified using full-length inactive DRONC (*Dronc^C318A^*). Western blot analysis to demonstrate binding specificity was carried out with 100 ng of full-length *Dronc^C318A^* and *Dronc^1–352^*. These were detected using purified anti-DRONC peptide (1:100) or purified anti-active DRONC peptide (1:100) antibodies.

#### Male fertility tests

Individual male flies were placed with 4- to 5-d-old virgin females in vials for 3 d at 25°C. They were then transferred to fresh vials with four new females and allowed to mate for another 3 d. Males were scored as sterile if they failed to produce progeny by day 6.

#### Western blotting of adult testis

Testes extracts were prepared in 50 μl of cell lysis buffer (20 mM HEPES–KOH [pH 7.6], 150 mM NaCl, 10% glycerol, 1% Triton X-100, 2 mM EDTA, 1× protease inhibitor cocktail [Roche, Basel, Switzerland], and 1 mM DTT) from 30–50 adults of the appropriate genotype. Total protein (70 μg) was used for Western blot analysis using rabbit anti-ARK (1:1,000) (generously provided by Lai Wang and Xiaodong Wang) or purified rabbit anti-DCP-1 (1:100). Filters were stripped using Restore Western blot stripping buffer (Pierce Biotechnology, Rockford, Illinois, United States) and reprobed with rabbit anti-full-length DRICE (1:1,000) (Dorstyn et al. 2002) as a loading control.

#### Electron microscopy

Testes were dissected from adult 2- to 4-d-old males raised at 25°C and prepared for EM as described by Tokuyasu et al. (1972). Thin sections were observed and photographed using a Philips 201 transmission electron microscope (Royal Philips Electronics, Eindhoven, The Netherlands) at 80 kV accelerating voltage. Elongated cysts in which spermatids should have been undergoing or have undergone individualization were identified by their central position in the testis as well as the stage of differentiation of major and mitochondrial derivatives (Tokuyasu et al. 1972). At least two to three testes of each genotype were examined.

## Supporting Information

Figure S1 Inhibition of *Dcp-1* Prevents Spermatid Individualization (A) EM section from β2tub-Dcp-1-RNAi testis. Individualization has failed to occur throughout the cyst.
(B) The boxed area in M is shown at a higher magnification. Spermatid units sharing a common cytoplasm are outlined by the dashed line.
(C) Western blot from wild-type (Wt) and β2tub-Dcp-1-RNAi (Dcpi) testis probed with anti-DCP-1 and anti-DRICE antibodies. DCP-1, but not DRICE, levels are greatly reduced in β2tub-Dcp-1-RNAi testis.
(552 KB JPEG).

Figure S2 Antibodies Specific for Active DRONC (A) Third instar eye imaginal disc from *GMR-Dronc^ F118E^* larvae stained with purified anti-DRONC peptide antiserum (green). All cells posterior to the morphogenetic furrow labeled with this antiserum, as expected based on the pattern of GMR (glass multimer reporter)-dependent gene expression (Hay et al. 1994). Eye discs from wild-type larvae showed only very low, uniform levels of staining (data not shown). The inset shows a Western blot probed with purified anti-DRONC peptide antiserum. The first lane was loaded with full-length DRONC mutated in its active site (*Dronc^C318A^*). The second lane was loaded with a version of DRONC consisting of only residues 1–352. This protein terminates following glutamate-352, the DRONC autoactivation cleavage site, and is equivalent to the large subunit of cleaved and active Dronc. The anti-DRONC antibodies react well with both proteins.
(B) Third instar eye imaginal disc from *GMR-Dronc ^F118E^* larvae stained with anti-active DRONC antiserum extensively purified to select for antibodies that react only with versions of DRONC that have been cleaved at glutamate-352, as described in the Materials and Methods. Only cells in the most posterior region of the eye disc, which are presumably undergoing apoptosis, react with these purified antibodies. The inset shows a Western blot, similar to that in (A), which was probed with the purified active DRONC-specific antibodies. These antibodies react with the glutamate-352-cleaved version of DRONC, but not with full-length DRONC.
(374 KB JPEG).

### Accession Numbers

The National Center for Biotechnology Information (NCBI) (http://www.ncbi.nlm.nih.gov/) accession number for p35 is P08160.

The FlyBase (http://flybase.bio.indiana.edu/search/) accession numbers of the sequences discussed in this paper are *Ark* (FBgn0024252), cyt-c-d (FBgn0000408), *Dcp-1* (FBgn0010501), *dFadd* (FBgn0038928), *Diap1* (FBgn0003691), *Dredd* (FBgn0020381), *Drice* (FBgn0019972), *Dronc* (FBgn0026404), *Grim* (FBgn0015946), *Hid* (FBgn0003997), and *Reaper* (FBgn0011706).

## Abbreviations

β2tub: β_2_-tubulin
cyt-c-d: cytochrome c-d
DCP-1: *Drosophila* caspase-1
Dn: dominant negative
EM: electron microscopy
GMR: glass multimer reporter
IAP: inhibitor of apoptosis
IMD: immune deficiency
PGRP: peptidoglyclan recognition protein
RNAi: RNA interference
