# Supplementary figures and images for: Multiple Apoptotic Caspase Cascades Are Required in Nonapoptotic Roles for Drosophila Spermatid Individualization
Source: PLoS Biol. 2003 Dec 15;2(1):e15. doi: 10.1371/journal.pbio.0020015 (PMC300883; doi:10.1371/journal.pbio.0020015)

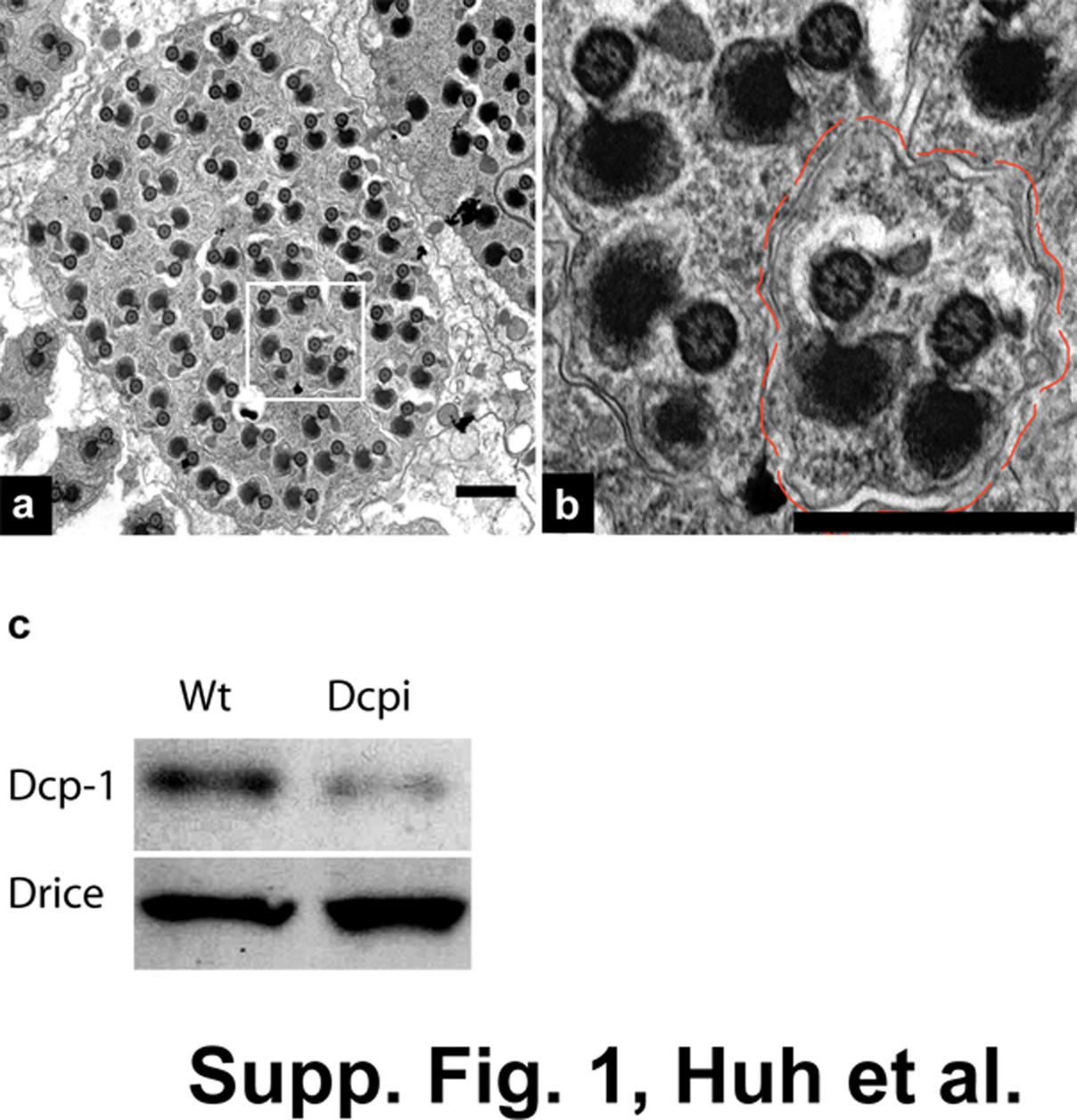

Supplement: Figure S1 — (A) EM section from β2tub-Dcp-1-RNAi testis. Individualization has failed to occur throughout the cyst. (B) The boxed area in M is shown at a higher magnification. Spermatid units sharing a common cytoplasm are outlined by the dashed line. (C) Western blot from wild-type (Wt) and β2tub-Dcp-1-RNAi (Dcpi) testis probed with anti-DCP-1 and anti-DRICE antibodies. DCP-1, but not DRICE, levels are greatly reduced in β2tub-Dcp-1-RNAi testis. (552 KB JPEG). [file pbio.0020015.sg001.jpg]

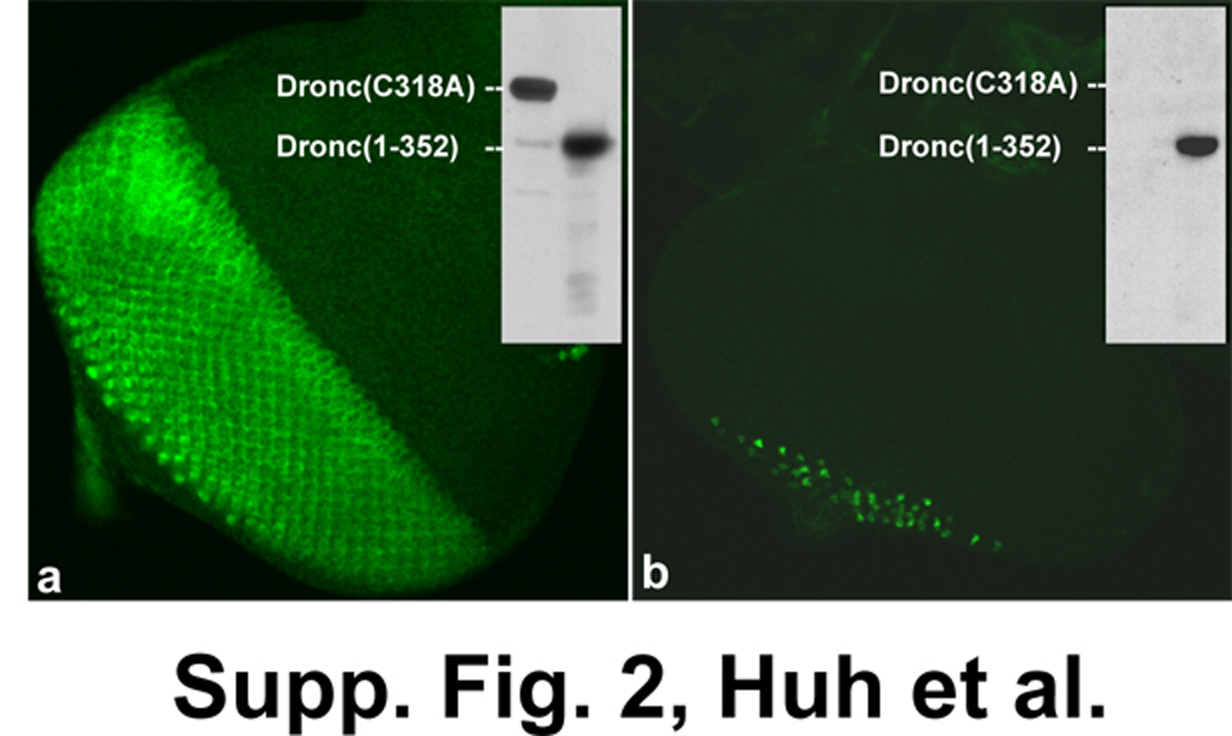

Supplement: Figure S2 — (A) Third instar eye imaginal disc from GMR-Dronc F118E larvae stained with purified anti-DRONC peptide antiserum (green). All cells posterior to the morphogenetic furrow labeled with this antiserum, as expected based on the pattern of GMR (glass multimer reporter)-dependent gene expression (Hay et al. 1994). Eye discs from wild-type larvae showed only very low, uniform levels of staining (data not shown). The inset shows a Western blot probed with purified anti-DRONC peptide antiserum. The first lane was loaded with full-length DRONC mutated in its active site (DroncC318A). The second lane was loaded with a version of DRONC consisting of only residues 1–352. This protein terminates following glutamate-352, the DRONC autoactivation cleavage site, and is equivalent to the large subunit of cleaved and active Dronc. The anti-DRONC antibodies react well with both proteins. (B) Third instar eye imaginal disc from GMR-Dronc F118E larvae stained with anti-active DRONC antiserum extensively purified to select for antibodies that react only with versions of DRONC that have been cleaved at glutamate-352, as described in the Materials and Methods. Only cells in the most posterior region of the eye disc, which are presumably undergoing apoptosis, react with these purified antibodies. The inset shows a Western blot, similar to that in (A), which was probed with the purified active DRONC-specific antibodies. These antibodies react with the glutamate-352-cleaved version of DRONC, but not with full-length DRONC. (374 KB JPEG). [file pbio.0020015.sg002.jpg]
